# The Causation, Cure, and Treatment of Reflex Insanity in Women

**Published:** 1871-05

**Authors:** 


The Causation, Cure, and Treatment of Reflex Insanity in Women. By Horatio R. Storer, M.D., LL.B. Lee & Shepard, publishers, Boston.

This is a reprint of a communication made by Prof. Storer to the American Medical Association, at its meeting in Boston, in 1855, and published in the Transactions of the Association for that year. It forms a small volume of 236 pages; well worthy of the perusal of the general practitioner, and of special interest to those engaged in the management of female diseases, whether mental or physical.

